# A Comprehensive High-Quality DNA and RNA Extraction Protocol for a Range of Cultivars and Tissue Types of the Woody Crop Avocado

**DOI:** 10.3390/plants11030242

**Published:** 2022-01-18

**Authors:** Onkar Nath, Stephen J. Fletcher, Alice Hayward, Lindsay M. Shaw, Rimjhim Agarwal, Agnelo Furtado, Robert J. Henry, Neena Mitter

**Affiliations:** The Queensland Alliance for Agriculture and Food Innovation, The University of Queensland, Brisbane 4072, Australia; o.nath@uq.edu.au (O.N.); s.fletcher@uq.edu.au (S.J.F.); a.hayward@uq.edu.au (A.H.); lindsay.shaw@uq.edu.au (L.M.S.); rimjhim.agarwal@uq.edu.au (R.A.); a.furtado@uq.edu.au (A.F.); robert.henry@uq.edu.au (R.J.H.)

**Keywords:** DNA extraction, RNA extraction, avocado, CTAB, tissues, cultivars, woody species

## Abstract

High-quality DNA and RNA forms the basis of genomic and genetic investigations. The extraction of DNA and RNA from woody trees, like avocado (*Persea americana* Mill.), is challenging due to compounds which interact with nucleic acids and influence separation. Previously reported methods of DNA and RNA extraction from avocado have issues of low yield, quality and applicability across different cultivars and tissue types. In the current study, methods have been optimised for high-quality DNA extraction from 40 avocado cultivars and RNA extraction from multiple tissue types, including roots, stem, leaves, flowers and fruits. The method is based on the modification of the cetyltrimethylammonium bromide buffer, centred around the specific optimisation of chemicals, such as sodium dodecyl sulphate, polyvinylpyrrolidone, sodium sulphite, polyethylene glycol and β-mercaptoethanol. The DNA extraction method yielded high-molecular weight DNA from the leaf tissue of 40 avocado cultivars belonging to Mexican, Guatemalan and West Indian avocado horticultural groups. The method was further optimised for RNA extraction from different avocado plant parts, enabling extraction using amounts as low as ~10 mg of starting material. The DNA and RNA extracted was successfully used for long- and short-read sequencing and gene expression analysis. The methods developed may also be applicable to other recalcitrant plant species.

## 1. Introduction

Deciphering molecular and cellular mechanisms using common molecular biology techniques, such as polymerase chain reaction (PCR), quantitative polymerase chain reaction (qPCR), RNA-Seq (RNA sequencing), cDNA (complementary DNA) library synthesis, gene cloning and northern blot analysis requires the use of high-quality nucleic acids. In addition, long-read sequencing techniques require high-molecular weight, contaminant-free DNA for the generation of long contigs and full-length transcripts [1,2]. However, the extraction of nucleic acids of suitable quality for current sequencing technologies from woody, recalcitrant plant species has proven to be difficult.

Avocado (*Persea americana* Mill.) is a high-value, rapidly growing horticultural crop, with production having increased by 34.8% since 2015 to 7.2 million metric tons annually [3]. Avocado comprises three major horticultural groups, namely, Mexican, Guatemalan and West Indian, corresponding to the three geographical areas of highland Mexico, highland Guatemala and lowland Mexico, respectively [4,5,6,7]. Hass, a Mexican and Guatemalan hybrid, remains the most important commercial variety [8]. With the rapid expansion of the avocado industry, advances in molecular biology and genomic resources are important for the improvement of varieties.

Avocado tissues are rich in fats, carbohydrates, fibres like cellulose, polysaccharides, proteins, lipids and secondary metabolites, including polyphenols [9,10,11]. These can act as contaminants that hinder the extraction of nucleic acids essential for downstream molecular analyses in plants [12,13]. Many have ionic similarity or compatibility to nucleic acids; thus, they co-precipitate [14,15], whereas others react with various compounds during the process. Phenolic compounds, which are oxidised after cell lysis, allowing them to bind to nucleic acids irreversibly, co-precipitate and make nucleic acids challenging to dissolve [16,17,18,19,20,21,22]. Contaminants can also impact yield and the accurate quantification of nucleic acids and can inhibit enzymes in downstream applications, including PCR and library preparation processes for nucleic acid sequencing. For example, they can interact with enzymes, such as polymerases, creating nicks, abasic sites or a DNA adduct, affecting the library insert size and the resources required for long-read sequencing [23,24]. In particular, the ability to obtain ultra-long reads is severely impacted, impeding high-quality genome and transcriptome assemblies. Contaminants may also result in the degradation of the extracted DNA [25,26].

RNA was first reported to be extracted from avocado by Christoffersen et al. [27]. They extracted mRNA for gene expression analysis from Hass fruit tissues shortly after harvest at the unripe and ripe stage using CTAB (cetyltrimethylammonium bromide) and phenol purification steps. Djami-Tchatchou et al. [28] later reported a CTAB-based method for high-quality RNA extraction from Fuerte avocado fruits without using phenol. RNA quality was assessed by spectrophotometric analysis and reported to be suitable for cDNA synthesis. More recently, Barbier et al. [29] reported a phenol/chloroform-free, CTAB/SDS based protocol that yields high RNA concentrations from limited tissue types (stem, leaf, flower and axillary buds) from Hass fruits. They further optimised this method for extraction of samples in a 96-well plate format. This protocol enabled quantification of miRNA expression and the generation of a transcriptome using short-read sequencing from macadamia, avocado and mango. Barbier et al. [29] also reported the adaptability of their RNA protocol as a DNA extraction method. Rendon-Anaya et al. [30] sequenced and analysed the nuclear genomes of Hass and Mexican variety *drymifolia* and re-sequenced the genomes of cultivars Carmen Hass, Velvick and the varieties *costarricensis*, *guatemalensis* and *americana*. For Hass and *drymifolia* reference genomes, they first isolated nuclei from young expanding leaves using a protocol by Steinmuller et al. [31], then isolated high-quality megabase-sized DNA using a method established by Hanania et al. [32]. The extracted DNA was successfully used for 80× long-read PacBio sequencing and Illumina sequencing.

The development of different protocols specific to tissues or cultivars is not desirable for molecular analysis as it imposes handling difficulties in experiments and significantly lengthens the time needed to process samples. To date, published DNA and RNA extraction protocols for avocado have been reported for only a few tissue types and cultivars. Differences between different tissue types as well as cultivars has made it difficult to develop an all-in-one protocol. Here we report an improved CTAB-based protocol to extract long-read sequencing quality DNA from both young and mature leaves from 40 different avocado cultivars. This method efficiently eliminates contaminants that inhibit downstream processes and does not involve the isolation of nuclei required in previous sequencing publications to extract minimally fragmented DNA. Similarly, a modified CTAB/SDS (sodium dodecyl sulphate)-based RNA extraction protocol has been developed to obtain a high quality and quantity of RNA from all major tissue types in avocado, including roots, callus, shoots, flowers, fruits, seeds and leaves throughout development. These protocols have been used successfully for long-read sequencing that demands high-molecular weight, intact and contaminant-free DNA and total RNA, as well as short-read sequencing, PCR and qPCR experiments. These DNA and RNA extraction protocols have also been successfully applied with additional plant species, including capsicum, cotton and finger lime (data not shown).

## 2. Results

### 2.1. DNA Extraction

DNA was extracted from the leaf tissue of 40 avocado cultivars (see Table 1) representing the three horticultural groups of avocadoes, West Indian, Guatemalan and Mexican, which are known to vary widely in their traits and morphologies. These cultivars included rootstocks and scions and A and B flowering varieties. The reported DNA extraction protocol was conceptualized based on previous extraction protocols by Singh et al. [33], Zou et al. [34], Xia et al. [35] and Barbier et al. [29] and optimised for avocado. Concentrations of EDTA, salts, sodium sulfite (Na_2_SO_3_), PVP (polyvinylpyrrolidone), incubation times and temperatures were optimised.

CTAB lysis buffers with high salt concentrations have been developed and regularly used for recalcitrant tree species, as most commercial kits produce very low yields, if any, in avocado [36,37,38]. We therefore used CTAB as a lysing agent in our buffer. Phenol-based lysis buffers from Schmitt et al. [39] and Chomczynski et al. [40] were tested and were not found optimal in terms of yield and quality. The most likely reason may be the presence of bioactive phytochemicals, like phenolic acids, in avocado tissues. [41].

Generally, CTAB-based protocols suggest using a high amount of initial tissue (1 g/10 mL reaction or 0.3 mg /mL) [42,43], but in the case of avocado, using 0.5 g of tissue per 10–20 mL reaction gave a better result in terms of concentration and quality of extracted nucleic acids. The method presented here yielded a high quantity and concentration of DNA from 1 g of plant tissue for other plants, including capsicum (Supplementary Material Figure S1), cotton and finger lime (data not shown), indicating the possible presence of compounds in avocado that saturate the buffer capacity. For some cultivars (particularly the samples from the USA shipped in CTAB buffer), OB2 buffer was required, as the extracted DNA concentration was low. The OB2 buffer adds extra salt to the solution, providing stability and increasing the precipitation rate at the cost of high salt co-precipitants at the end of the protocol.

Avocado leaves contain a high concentration of phenols, saponins, flavonoids and steroids [44]. Avocado is also rich in proteins, structural polysaccharides, fibres and ash [45,46]. The concentration of potassium, sodium, magnesium, calcium and phosphorous is also high [47,48,49]. Some of these compounds hinder nucleic acid extraction by either interacting with the buffer or precipitating with the DNA or by collating into the DNA strand [50]. Adding MES (2-(N-morpholino) ethanesulfonic acid), PEG (polyethylene glycol) and Na_2_SO_3_ to the buffer is suggested to help eliminate these compounds [18,51,52]. High levels of co-precipitated contaminants, such as polyphenols and tannins, have been shown to be removed using the antioxidant PVP and reducing agent 2ME (β-mercaptoethanol) [53,54]. Adding the reducing agent sodium sulfite plus PVP can stabilise oxidised phenols, assisting the purification of intact DNA [52,55]. Sodium sulfite and 2ME are also reported to increase the quality of the DNA [18,51,52].

High levels of oxidised phenolics, similar to those found in avocado, are well known to hinder DNA and RNA extraction [16,17,18,19,20,21,22]. While phenol:C:IAA (phenol:chloroform:isoamyl alcohol) is commonly used during CTAB extraction, the DNA degrades in the case of avocado. Here, C:IAA alone, added at least twice before precipitating using cold isopropanol, was found to be effective.

**Figure 1:**
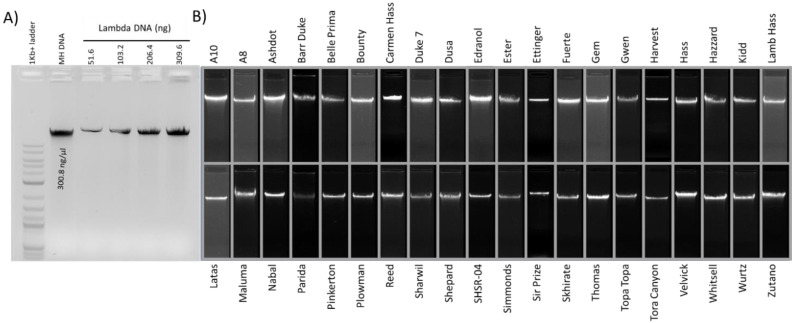
Intact DNA from avocado genotypes as indicated by agarose gel electrophoresis. (**A**) Concentration of Hass avocado DNA (MH DNA) calculated using standard lambda DNA concentrations with Gel DocTM XR+ with Image LabTM software. (**B**) Composite image corresponding to 40 genotypes indicating intact DNA.

The DNA extraction method developed here is simple and yields good quality intact DNA from 40 different avocado cultivars with a 260 nm/280 nm absorbance ratio of, on average, 1.8 (1.45–2.03) (see Table 1) and a 260 nm/230 nm absorbance ratio that varied between 0.75–1.83, depending on the cultivar. The purity, quality and quantity of extracted DNA from leaf samples, reported in Table 1 and Figure 1, are acceptable for downstream applications, as detailed further below.

### 2.2. Sequenced DNA Read Statistics

**Figure 2:**
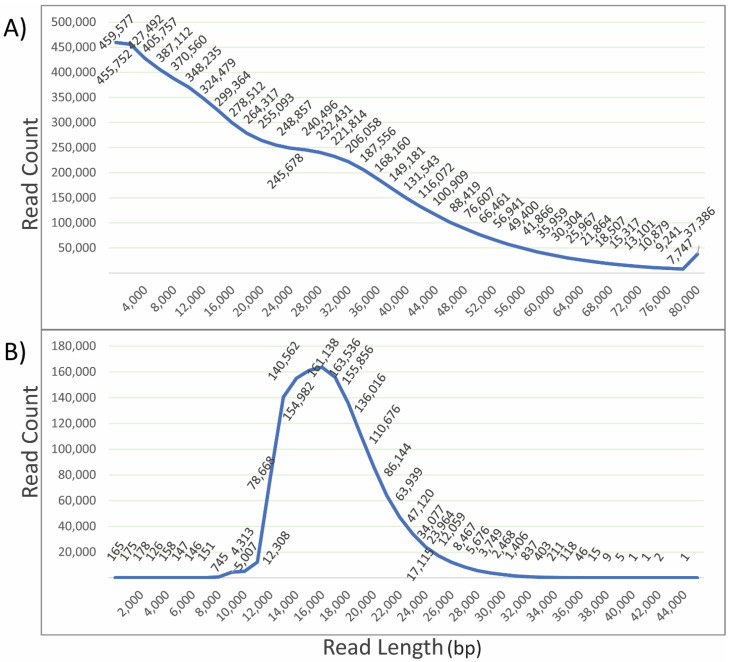
PacBio long- and HiFi-sequenced read length distribution vs. read count for Hass avocado. (**A**) Long read length distribution depicts a large number of reads of short length, i.e., below 10 kb length. However, a significant number of reads were present for long and ultra-long reads. (**B**) HiFi read count (peak at 16 kb) with respect to read length, which corresponds to the prepared library, indicating the least sequencing bias.

**Figure 3:**
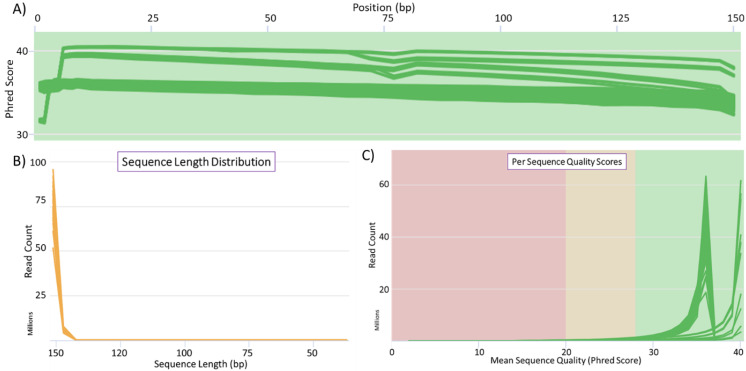
Raw read quality statistics for 40 avocado cultivars sequenced using the Illumina platform. (**A**) Mean Quality Score plot: indicates high quality sequenced and filtered bases, as the mean score plot lies in the quality passed read (green) region above Phred score 30. (**B**) Per Sequence Quality Scores: indicate that most of the sequences have a quality score above 30. (**C**) Length distribution: this length distribution graph indicates that most of the reads generated were of full length, i.e., about 150 bases.

Input DNA quality is crucial for sequencing and determines the quality of sequence data. The extracted DNA was sequenced using PacBio long-read sequencing, PacBio HiFi sequencing and Illumina short-read sequencing (Figure 2 and Figure 3). The length distribution of sequenced reads indicates a large number of reads with lengths greater than 80 kb. However, a very high number of reads were obtained with lengths below 25 kb. Shorter reads are generally preferentially sequenced, which leads to a higher proportion of short reads over long reads. This can be controlled in HiFi sequencing during library preparation; thus, a very high number of reads were obtained of a length around 17 kb. The quality statistics analysis using FastQC (http://www.bioinformatics.babraham.ac.uk/projects/fastqc/, accessed on 22 December 2021) for Illumina-sequenced DNA samples indicated a high Phred score of the sequenced reads and corresponding bases (Figure 3). Phred score is a scoring and calling system used to measure the quality of nucleotide calls in sequencing [63]. Bases and reads with a quality score above 30 are considered high quality. In our case, all the reads were within an acceptable range for quality, as were most of the bases. The length of the sequenced reads was around 150 bases. The pulse-field DNA length distribution of the extracted DNA indicated that the extracted DNA was of high molecular weight. This confirmed that the DNA extracted can be successfully used for sequencing purposes using available long- and short-read sequencing platforms for genetic and genomic analysis.

### 2.3. Downstream DNA Analysis

**Figure 4:**
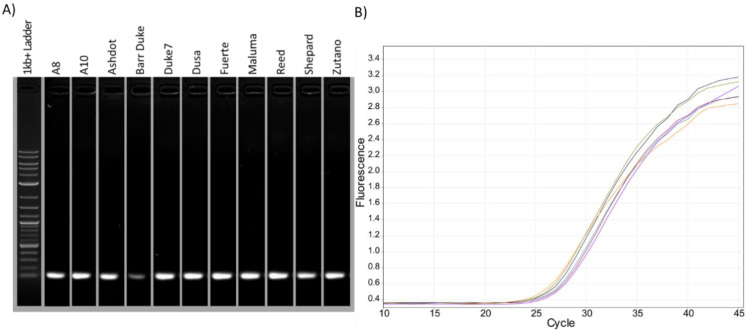
PCR and qPCR amplification using genomic DNA extracted from leaves of various cultivars. (**A**) Intact single band of the amplified PCR primer products from different cultivars on agarose gel. (**B**) Corresponding qPCR amplification curve.

The quality of extracted DNA and RNA plays a vital role in the success of amplification [64]. Contaminants may interact with the polymerase and inhibit its activity or may chelate with nucleic acids, imposing binding competition with various components of extraction buffer. To further confirm whether the quality of the extracted DNA was suitable for common molecular biology processes, PCR and qPCR were performed using primers corresponding to contig SDSS01000523.1 at position 285719 [30] from multiple cultivars (Figure 4). PCR products were amplified from all cultivars tested at the expected size of 106 bp when run on a gel (Figure 4A) and expected amplification curves when using qPCR (Figure 4B).

### 2.4. RNA Extraction

Several different tissues (at various developmental stages) of Hass avocado were collected for extracting total RNA. Variation in properties of different avocado tissues [65,66,67,68] is hypothesized here as a limiting factor in developing a one-for-all extraction protocol. Here a protocol has been developed to successfully extract RNA from various tissues (see Table 2). Although the protocol worked well with all the tissues, ripe fruit pulp and roots were difficult to extract. In these cases, subsequent purification processes were necessary to allow RNA resuspension in the solution (see Section 3).

The amount of tissue used was crucial. Here the use of 0.01 g (10 mg) per 1.5 mL reaction to obtain a high concentration and quality of extracted nucleic acids is recommended. For seeds, more tissue (i.e., 50 mg instead of 10 mg) yields more nucleic acids. However, for roots, more tissue led to degradation of the 5S ribosomal RNA band. Most other tissues did not support high quantities (>10 mg/mL) of starting material. When tested with capsicum (Supplementary Material Figure S1), finger lime and cotton (data not shown), 50 mg tissue yielded higher concentration with good quality RNA. This may indicate the presence of some compound/s in avocado that saturates the buffer capacity.

The incubation time also varied for some tissues. Unripe fruit seeds required a very long incubation time (30 min); in contrast, roots could not withstand more than 5 min of incubation at 65 °C. Leaves yielded RNA at various time and temperature ranges but had maximum efficiency between 10–25 min at 65 °C.

CTAB was used as lysis agent in OB1-R for developing the current protocol. SDS buffers also assist nucleic acid extraction but they are not preferred, as they co-precipitate proteins. Xia et al. [35] and Barbier et al. [29] demonstrated the use of SDS surfactant as a lysis agent. It was found here that using SDS and sodium sulfite reduces degradation, which was an issue with certain tissues; thus, a low concentration (0.1% *v*/*v*) of SDS to assist lysis but to avoid protein contamination was used. The percentage of SDS used was increased (1% *v*/*v*) in the case of capsicum fruit (Supplementary Material Data S1).

The composition of OB1-D and OB1-R for DNA and RNA extraction, respectively, is mainly similar. Sodium salts, PVP, 2ME and SDS in the buffers were used to promote cell lysis and prevent RNase activity and polyphenols from co-precipitating [69]. It was found that use of OB2 buffer, which comprises salts, such as sodium citrate, N-Lauroylsarcosine sodium salt and sodium acetate, reduces RNA degradation [70]. Guanidine thiocyanate, the additional option in the OB2 buffer, was included to stabilize RNA by denaturing proteins [40,71]; however, it encouraged solidification in starch-rich samples, like seeds [72]. In addition, a clean-up step using SSTE buffer was performed in samples where a starchy solidified precipitant was observed.

This method yielded sizes ranging from small RNAs to ribosomal and large mRNAs. pH played a significant role in buffers. The optimal pH for OB1-R was 7.6, except for unripe fruit seeds, which worked best at pH 6. In the case of leaves, a high total RNA concentration was obtained at pH 6 but appeared to precipitate higher quantities of smaller RNAs (based on 5S RNA content, i.e., 120 nucleotides as visualised on the gel). Intact ribosomal RNA could not be isolated at pH 8 or 6.5. This pH range also fits with the recommended pH exposure range (6–9) by Pacific Bioscience for long-read sequencing [24]. Isopropanol was used for precipitation of both DNA and RNA, as reported by Aboul-Maaty et al. [73] and Barbier et al. [29]. Double precipitation using LiCl improves quality, but as LiCl size selects RNA [74,75], it is not ideal for total RNA extraction. It is thought that the RNAs, like tRNAs, are not efficiently precipitated with LiCl due to the high degree of secondary structure [76].

The extracted RNA samples were analysed for integrity, quality and quantity using the NanoDrop spectrophotometer, Quantifluor^®^ fluorometer (Promega, Madison, Wisconsin, USA), Bioanalyzer (Agilent Technologies, Santa Clara, CA, USA) and agarose gel electrophoresis (Bioline, London, UK). Samples (leaf, root, fruit seed and fruit pulp) that failed to pass the cut-offs (i.e., 260/230 and 260/280 ratios 1.6 ≥ 2) were cleaned using additional methods. Firstly, column-based methods (RNeasy^®^ Mini Kit and RNeasy^®^ Plant Mini Kit from QIAGEN, Hilden, Germany [77] and ISOLATE II RNA Plant Kit from Bioline, London, UK) were trialled; however, they couldn’t precipitate and clean the RNA efficiently. With columns, two significant issues were encountered: first, the recovery rate was consistently below 10 per cent of the initial RNA concentration; secondly, the smaller RNAs were severely impacted, as the 5.8 and 5S (250 bp) band was very faint or was missing after clean-up. Instead, the XP clean RNA beads from Beckman Coulter, Brea, California, USA were valuable to clean the RNA.

**Figure 5:**
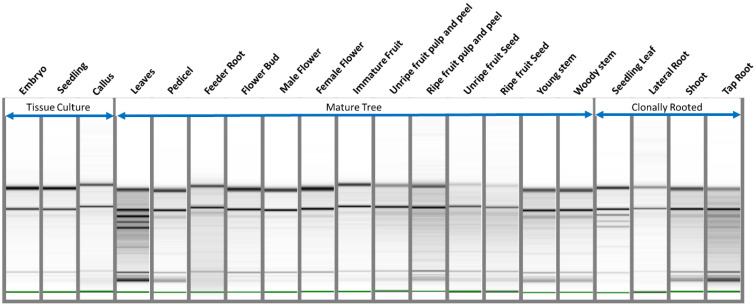
Intact RNA bands, of high quality and quantity, for various Hass tissues visualized with Bioanalyzer gel. The gel image plotted by the Bioanalyzer indicates intact ribosomal RNA and other RNA bands. Details of tissues are reported in Supplementary Material Table S1.

For extracted RNA, the 260/280 ratios obtained using a spectrophotometer were between 1.37 and 2.21 and 260/230 ratios were between 0.57 and 2.4 (Supplementary Material Figure S2). The RIN numbers averaged around 7.5 (See Table 2), where 10 is considered to have the highest integrity. High RIN values along with adequate spectrophotometer ratios indicated that the extraction yielded high-quality and high-integrity RNA suitable for sequencing. The total RNA extraction method developed was successfully used for the long-read and short-read RNA sequencing of a pooled sample of different tissues of Hass avocado using the PacBio Iso-Seq Sequel II platform (Pacific Biosciences, Menlo Park, CA, USA) and Nova-Seq 6000 Illumina Sequencing (San Diego, CA, USA) (Figure 5).

### 2.5. Sequenced RNA Read Statistics

**Figure 6:**
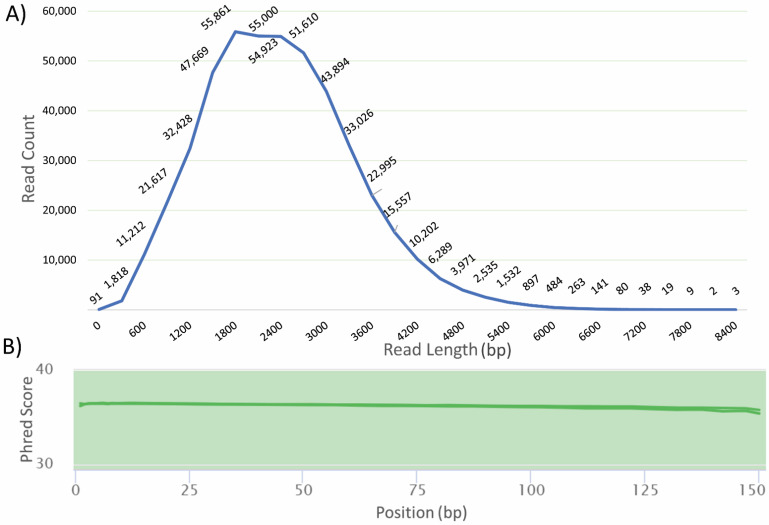
Read length and quality statistics of sequenced transcriptomes of pooled Hass tissues. (**A**) The length distribution of transcripts sequenced using Iso-Seq sequencing indicates the presence of long and short transcripts. (**B**) Mean quality score of RNA-Seq sequenced reads depicts a high Phred score for sequenced reads.

The input RNA quality is a major factor for obtaining high-quality sequenced reads. For the pooled RNA sequencing, the length distribution of the Iso-Seq reads ranged from 64 to 8510 nt, with an average read length of 1.8 kb. The length distribution is further suggestive of the protocol yielding total RNA (Figure 6A). Statistical quality analysis using FastQC for Illumina-sequenced RNA samples indicated a high Phred score, i.e., above 30, for the sequenced reads (Figure 6B). This confirms that the RNA extracted can be successfully used for sequencing purposes using available long- and short-read sequencing platforms for transcriptomic analysis.

## 3. Materials and Methods

### 3.1. Sample Collection and Processing

Leaves from 40 avocado cultivars (see Table 1) were collected for DNA extraction. These were sourced from the Department of Agriculture and Fisheries (DAF) avocado germplasm repository at the Maroochy Research Facility (Coes Creek, Queensland, Australia), Anderson Horticulture Pty, Ltd. (Duranbah, NSW, Australia), Fleming’s Nursery (Woombye, Queensland, Australia), Lynwood Avocado Nursery, Ltd. (Maunu, Whangārei, New Zealand) and the University of California, Riverside, USA. International samples were sent to Australia as ground samples in 2% CTAB buffer as per Australian biosecurity requirements. The sampling was carried out using mature avocado trees, and both new flush as well as mature leaves were pooled together for DNA extraction. Samples were immediately placed on dry ice and stored at −80 °C. For total RNA extraction, Hass avocado tissues across all major organs (leaves, flower buds, male and female flowers, immature fruit, unripe and ripe fruit pulp and peel, whole seed, soft-wood and hard-wood stems, feeder roots and embryos) were collected in liquid nitrogen from a mature tree at the DAF avocado germplasm repository (Supplementary Material Table S1). In addition, various tissues (whole seedling, leaves, stem, root and callus) were harvested from avocado seeds grown in tissue culture and glasshouse conditions [78]. Samples were pulverised in liquid nitrogen using a mortar and pestle or using metal beads in a TissueLyser II (QIAGEN, Hilden, Germany)) and stored at −80 °C before use in extraction.

### 3.2. Nucleic Acid Extraction Buffers

#### 3.2.1. Optimised Buffer 1-D (OB1-D) (DNA Extraction) (pH 7.6)

Optimised Buffer 1-D contains 2% CTAB, 1.4M NaCL, 10 mM EDTA, 0.1M MES, 1% PEG (filter sterilized buffer). At the time of use, 0.5% Na_2_SO_3_, 2% PVP, 2% β-mercaptoethanol (2ME) are to be added. For samples with high co-precipitants, up to 5% of 2ME can be used. Adjust the pH to 7.6 using NaOH.

#### 3.2.2. Optimised Buffer 1-R (OB1-R) (RNA Extraction) (pH 7.6)

Optimised Buffer 1-R contains 2% CTAB, 1.4M NaCL, 10 mM EDTA, 0.1M MES, 1% PEG (filter sterilized buffer). At the time of use, 0.01% *v/v* of 10% SDS and 2% β-mercaptoethanol are to be added. Adjust the pH to 7.6 using NaOH.

#### 3.2.3. Optimised Buffer 2 (OB2)

Optimised Buffer 2 contains 25 mM sodium citrate, 0.5% N-lauroylsarcosine sodium salt, 1 M sodium acetate, 2% PVP and 4.2 M guanidine thiocyanate (optional).

#### 3.2.4. Optional Clean-Up Buffer (SSTE) 

The optional clean-up buffer contains 1 M NaCl, 0.5% SDS, 10 mM Tris (pH 8.0) and 1 mM EDTA (pH 8.0) [79].

#### 3.2.5. Other Reagents

The other reagents used were: chloroform:isoamyl alcohol (C:IAA) 24:1; isopropanol (100%); sodium dodecyl sulfate (SDS) (10%) (to be used within two months, as its efficiency reduces with time).

*appropriate PPE including lab coat and other safety apparatus is recommended for performing these protocols

### 3.3. DNA Extraction Protocol

There are two versions of this protocol, i.e., for a large (10–20 mL) volume (Protocol A) and a small (less than 2 mL) volume (Protocol B).

PROTOCOL A

1. Pulverise frozen tissue to powder under liquid nitrogen without the addition of buffer.
2. Add approximately 0.2 g of ground frozen leaf tissue to 15 mL hot OB1-D (65 °C) in a 50 mL tube and incubate at 60–65 °C for 5 min.
3. Centrifuge at 10,000 rpm (12,857 g) for 10–15 min at room temperature to pellet debris.
4. Transfer the supernatant to a new 50 mL tube and add 10 µL of RNase A (10ng/µL).
5. Incubate at 37 °C for 20–30 min. (This incubation is sufficient due to the high pH (7.6) of the buffer.)
6. Add an equal volume of C:IAA.
7. Mix the contents by gently inverting the tubes fifty times.
8. Centrifuge at 9500 rpm (11,603× *g*) at room temperature for 20 min.
9. Transfer the supernatant to a new 50 mL tube and repeat C:IAA wash again with half the total volume.
10. Transfer the supernatant to a new 50 mL tube and add an equal volume of cold 100% isopropanol (preferably at −20 °C or at 4 °C) and mix gently by inverting the tubes ten times. Precipitating with 95% ethanol can improve DNA quality with reduced yield for cultivars where more contaminant is precipitated.
11. Incubate at −20 °C for 30 min (can be left overnight). Extended incubation increases yield; however, it may increase salt content in the pellet.
12. Centrifuge at 10,000 rpm (12,857× *g*) for 25 min at 4 °C. Remove the supernatant.
13. Wash pellet with 7 mL 75% ethanol (or appropriate volume) to ensure the pellet is submerged. If needed, pool tubes at this stage to increase yield.
14. Centrifuge at 7500 rpm (7232× *g*) for 10 min. Centrifuge longer if pellet does not adhere to tube.
15. Carefully decant out the supernatant and air-dry the pellet, avoiding over-drying the pellet.
16. Dissolve the pellet in 50–200 µL of nuclease-free water or 1x TE buffer and store at −20 or −80 °C as required. DNA should be stable at room temperature for a few months.

Optional modifications: if the yield is low, add an equal volume of OB2 buffer at step 6 and proceed with the C:IAA wash. Adding this will reduce the 260/230 absorbance ratio.

PROTOCOL B

Follow the method for protocol A with the following modifications:

Step 2: Add approximately 10–30 mg of ground frozen leaf tissue to 750 µL hot OB1-D in a 2 mL tube and incubate at 60–65 °C for 5 min.

Steps 8,12: Centrifuge at 14,000 rpm (20,817× *g*) for the allotted times.

Step 4: Add 1 µL of RNase A (concentration 10 ng/µL).

Step 13: Wash pellet with 500 µL 75% ethanol.

Step 16: Dissolve the pellet in 20 µL nuclease-free water.

Notes: If high-quality avocado DNA is not required, a crude DNA extraction method that works well with leaf tissues can be used. For this, after the incubation at step 5, an equal volume of C:IAA is added and centrifuged at 14,000 rpm/21,000× *g*, followed by isopropanol precipitation and an ethanol wash.

### 3.4. RNA Extraction Protocol

1. Pulverise the frozen tissue to powder without the addition of buffer.
2. To OB1-R add 10 µL 10% SDS per 2ml buffer and 2% 2ME. Heat the buffer to 65 °C.
3. Add approximately 10 mg of ground frozen tissue to 650 µL hot OB1-R buffer in a 2 mL tube and incubate at 60–65 °C for 5 min. (Over-incubation leads to RNA degradation, particularly in root tissues. Further tissue-specific recommendations for improved quality are suggested in the results section.)
4. Centrifuge at 14,000 rpm (20,817× *g*) for 10 min at room temperature and transfer supernatant to a new tube.
5. Add an equal volume of OB2 buffer.
6. Add an equal volume of C:IAA.
7. Vortex tubes well.
8. Centrifuge at 14,000 rpm (20,817× *g*) at room temperature for 20 min.
9. Transfer supernatant to a new tube and repeat the chloroform wash.
10. For total RNA extraction, add an equal volume of 100% isopropanol (cold) and incubate at −20 °C for 1 h or longer as needed. Longer incubation time increases yield but also increases salt concentration in precipitate. Alternatively, use a half volume of 10M lithium chloride (LiCl) instead of isopropanol and incubate overnight at 4 °C. This can improve RNA quality, however, it may result in the loss of small and complex RNAs.
11. Centrifuge at 10,000 rpm (12,857× *g*) for 25 min at 4 °C.
12. Wash pellet with 1ml of 75% ethanol.
13. Centrifuge at 8500 rpm (7674× *g*) for 10 min.
14. Air dry the pellet, being careful to not over-dry, and dissolve the pellet in 20 µL of nuclease-free water.

#### DNase Treatment and Clean-Up

15. Samples can be pooled at this stage for higher RNA concentration.
16. If DNA must be removed from the RNA sample, proceed with DNase treatment by adding 0.05% *v/v* of DNase 1 buffer (New England Biolabs, Ipswich, Massachusetts, USA) and 0.05% *v/v* (24 units) of DNase 1 enzyme and incubate at 37 °C for 30 min.
17. Add DNase- and RNase-free water to make up 500 µL of total volume.
18. Add an equal volume of TRIsure™ (Bioline, London, UK) (use SSTE buffer if RNA degrades with the use of TRIsure™) and incubate for 3–5 min.
19. Add 100 µL chloroform and centrifuge at 14,000 rpm (20,817× *g*) for 20 min.
20. Transfer supernatant to a new tube and add an equal volume of isopropanol.
21. Incubate at −20 °C for a minimum of one hour.
22. Centrifuge at 14,000 rpm (20,817× *g*) for 40 min.
23. Wash pellet with ethanol, as described previously in steps 12–14.
24. Dissolve in 20 µL of nuclease-free water or 1× TE buffer and store at −20 or −80 °C.

Note: Tissue-specific recommendations are detailed in the results. If the following quality assessment of the RNA the pellet is still not clean, follow the RNAClean XP Bead protocol from Beckman Coulter using 100 µL buffer per 50 µL sample. Elute in nuclease-free water.

### 3.5. Quantification and Quality Control

Nucleic acid quality was checked on a 1% agarose gel and with capillary electrophoresis. Midori green stain (Nippon Genetics Europe GmbH, Düren, Germany) was added to agarose gels and visualized using the Gel Doc^TM^ XR+ with Image Lab^TM^ Software (Bio-Rad, Hercules, California, USA). The quantity of DNA was estimated using Gel Doc^TM^ XR+ with Image Lab^TM^ Software (Bio-Rad, Hercules, California, USA) by comparing band fluorescence intensities to that of standard lambda DNA quantity. The total amount was calculated using the formula: (relative concentration calculated using gel doc/volume loaded on gel) × total volume. The RNA quantity was estimated using an Agilent 2100 Bioanalyzer (Agilent Technologies, Santa Clara, California, USA). The purity was measured by absorbance at 230, 260 and 280 nm using a NanoDrop^®^ Spectrophotometer (Thermo Fisher Scientific, Waltham, Massachusetts, USA). In addition, RNA quality was estimated by the RIN value using an Agilent 2100 Bioanalyzer (Agilent Technologies, Santa Clara, California, USA).

PCR was performed using primers designed on contig SDSS01000523.1 at position 285719 of the avocado genome [30] (NCBI genome ID: ASM808724v1) (forward primer: TCTTTGCCTGTGCTGGTGAT; reverse primer: GCCATTCAAAGTCGTTGCCA). PCR was conducted in 20 µL reactions with 1X MyTaq reaction buffer (Bioline, London, UK), 1-unit MyTaq DNA polymerase, 10 µM primers and 100 ng of total DNA. PCR amplification was performed as follows: polymerase activation at 95 °C for 1 min, denaturation at 95 °C for 15 sec, followed by annealing at 60 °C for 15 s and finally extension at 72 °C for 10 s. This process was repeated for 35 cycles and fluorescence was acquired at the end of each step using a SimpliAmp Thermal Cycler (Thermo Fisher Scientific, Waltham, Massachusetts USA).

qPCR was performed in 20 µL reactions with 1X SensiFAST SYBR^®^ No-ROX mix (Bioline, London, UK), 400 nM primers and 50 ng of total DNA. qPCR was performed using the aforementioned primers as follows: polymerase activation at 95 °C for 3–5 min, followed by 45 cycles of denaturation at 95 °C for 5 s, annealing at 65 °C for 10 s and extension at 72 °C for 10 s. Melt curves were generated by melting from 65 to 95 °C, raising the temperature by 0.5 °C and waiting for 2 s after each step for clear fluorescence capture using a Rotor-Gene Q on Rotor-Disc 72, Hilden, Germany.

DNA was sequenced using PacBio long-read, PacBio HiFi-read and Illumina short-read sequencing techniques. For PacBio long-read sequencing, 20 µg genomic DNA was shipped to Nucleome Informatics Private Limited, India. Similarly, 20 µg genomic DNA was provided for HiFi sequencing at the Institute for Molecular Bioscience, University of Queensland, Australia. For Illumina nextra-seq sequencing and Illumina NovaSeq sequencing, respectively, using an S4 lane at an expected coverage of 20×, 2–4 µg genomic DNA was shipped to the Ramaciotti Centre for Genomics, Sydney, NSW, Australia and Macrogen, Seoul, South Korea. The RNA samples were pooled and sequenced using PacBio Iso-Seq sequencing and Illumina short-read sequencing. For Iso-Seq sequencing two libraries were prepared per sample (1 × standard workflow, 1 × long transcript workflow) using Iso-Seq Express library preparation and sequenced using 2 × PacBio Sequel II SMRT Cell 8M. Illumina reads were sequenced at an expected 100× coverage using NovaSeq.

## 4. Conclusions

Establishing an all-in-one protocol for challenging plants, like avocado, has proven difficult in the past. Here an efficient, high-quality DNA and total RNA extraction method for various avocado cultivars and tissue types has been reported. To our knowledge, this is the first report showing successful nucleic acid extraction from multiple tissues and cultivars of avocado in a comprehensive protocol. Recommendations established here could prove adaptable to extract high-quality nucleic acids from tissues from a range of difficult plant species.

## Figures and Tables

**Table 1:** Assessment of extracted DNA from leaf tissues of avocado cultivars. The 260/280 ratio and 260/230 ratios correspond to nanodrop values indicating DNA purity. Total amount of DNA extracted (column 5 and 10) refers to that calculated relative to lambda DNA standards. Samples marked US were obtained from UC, Riverside, USA. The Race column indicates horticultural groups, the “U”, “M”, “G” and “W” corresponding to unknown, Mexican, Guatemalan and West Indian, followed by corresponding references.

Cultivar	Race	260/280	260/230	DNA Concentration * (ug)	Cultivar	Race	260/280	260/230	DNA Concentration *(ug)
**A10**	G × M [56]	1.73	1.83	2.6	**Latas**	M [57]	1.99	1.67	2.4
**A8**	G [56]	1.96	1.54	1.7	**Maluma**	G × M [57]	1.95	1.71	4
**Ashdot**	W [56]	2.02	1.79	2.9	**Nabal**	G [56]	1.92	1.67	2.8
**Barr Duke**	M [56]	1.56	1.08	2.3	**Parida**	M [56]	1.45	1.66	2.3
**Belle Prima**	U	2.02	1.5	2.6	**Pinkerton**	G × M [58]	1.92	1.55	2.4
**Bounty**	M × G [57]	1.46	1.6	2	**Plowman**	G [56]	1.9	1.34	2.4
**Carmen Hass (US)**	G × M [57]	1.88	1.46	4.4	**Reed**	G [56]	1.96	1.66	2.8
**Duke7**	M [56]	1.7	1.52	1.8	**Sharwil**	M × G [57]	1.95	1.51	2.3
**Dusa**	G × M [57]	1.99	1.41	3.1	**Shepard**	M × G [56]	1.94	1.5	3.2
**Edranol**	G × M [56]	2.02	1.75	2.6	**SHSR-04**	G [59]	1.97	1.52	2.3
**Esther**	G [60]	2	1.77	3.1	**Simmonds**	W [60]	1.94	1.5	2.1
**Ettinger (US)**	M × G [61]	1.92	1.32	5.2	**Sir Prize (US)**	G × M [62]	1.6	0.75	2.4
**Fuerte**	M × G [60]	1.73	1.25	2.2	**Skhirate**	U	1.73	1.4	2
**Gem (US)**	G × M [57]	2.03	1.68	1.3	**Thomas**	M [56]	1.56	1.67	2.6
**Gwen**	G [60]	1.92	1.64	2.2	**Topa Topa**	M [60]	1.95	1.56	2.4
**Harvest (US)**	G × M [62]	1.96	1.35	3.6	**Tora Canyon**	M [56]	1.68	1.38	1.6
**Hass**	G × M [56]	1.96	1.25	1.9	**Velvick**	W × G [56]	1.94	1.8	3.2
**Hazzard**	G [60]	1.94	1.55	2.9	**Whitsell**	G [60]	1.71	1.49	2.6
**Kidd**	U	1.95	1.22	2.7	**Wurtz**	M × G [60]	1.77	1.47	2.1
**Lamb Hass**	G × M [57]	1.96	1.73	2.8	**Zutano**	M × G [56]	1.93	1.73	3.1

* Total DNA was extracted from 0.2 g of tissue for all cultivars collected in Australia and 0.4 g for those shipped from the USA.

**Table 2:** Samples for total RNA sequencing of Hass tissues and corresponding quality statistics. NanoDrop values and Bioanalyzer results indicate the quality of the extracted RNA. An RIN (RNA integrity number) above 6 is considered acceptable quality RNA; here in most cases, an RIN value above 6 and a very high concentration of total RNA were obtained. The concentration was calculated relative to an Agilent RNA 6000 Nano Ladder using a Bioanalyzer 2100 instrument (Agilent Technologies, Santa Clara, California, USA). Details of tissues are reported in Supplementary Material Table S1.

Tissue	260/280	260/230	RIN	Concentration (ng/μL)	Total Amount * (μg)
**Embryo**	2.08	1.83	8.6	579	11.58
**Seedling**	2.06	1	8.4	306	6.12
**Callus**	1.89	1.15	8	192	9.6
**Feeder root**	1.37	1.05	6.5	704	35.2
**Pedicel**	1.89	0.69	7.1	825	16.5
**Leaves**	2.08	1.73	6.4	2744	137.2
**Woody stem**	1.81	0.57	6.9	876	17.52
**Young stem**	1.93	0.9	6.4	1362	27.24
**Male flower**	1.97	0.93	8.5	1230	24.6
**Female flower**	2.03	1.15	7.7	2769	55.38
**Flower bud**	2.01	1.16	7.5	2097	41.94
**Unripe fruit pulp and peel**	2.16	1.23	6.8	576	28.8
**Ripe fruit pulp and peel**	2.1	1.74	5.9	2096	104.8
**Immature fruit**	2.01	2.17	7.3	5665	283.25
**Unripe fruit seed**	2.09	1.29	6	640	32
**Ripe fruit seed**	1.71	0.72	5.7	552	27.6
**Seedling leaf**	2.21	2.4	6.6	2568	128.4
**Lateral root**	1.87	0.92	7.8	112	5.6
**Shoot**	2.17	1.86	7.3	1028	51.4
**Tap root**	2.18	2.08	7.3	1408	70.4

* Total RNA extracted was from 0.01 to 0.03 g of frozen ground tissue.

## Data Availability

All data generated or analysed during this study are included in this published article.

## Supplementary Materials

The following are available online at https://www.mdpi.com/article/10.3390/plants11030242/s1, Data S1: Nucleic acids extracted from Capsicum, Figure S1: DNA and RNA extracted from the three capsicum fruit stages, Figure S2: Extracted RNA quality statistics for avocado, Table S1: Tissue collection details for the samples used for DNA and RNA extraction.
