# Supplementary material for: A Comprehensive High-Quality DNA and RNA Extraction Protocol for a Range of Cultivars and Tissue Types of the Woody Crop Avocado
Source: Plants (Basel). 2022 Jan 18;11(3):242. doi: 10.3390/plants11030242 (PMC8838124; doi:10.3390/plants11030242)
Supplement: Supplementary file 1 [file plants-11-00242-s001.zip › plants-1547302-supplementary/plants-1547302-supplementary]

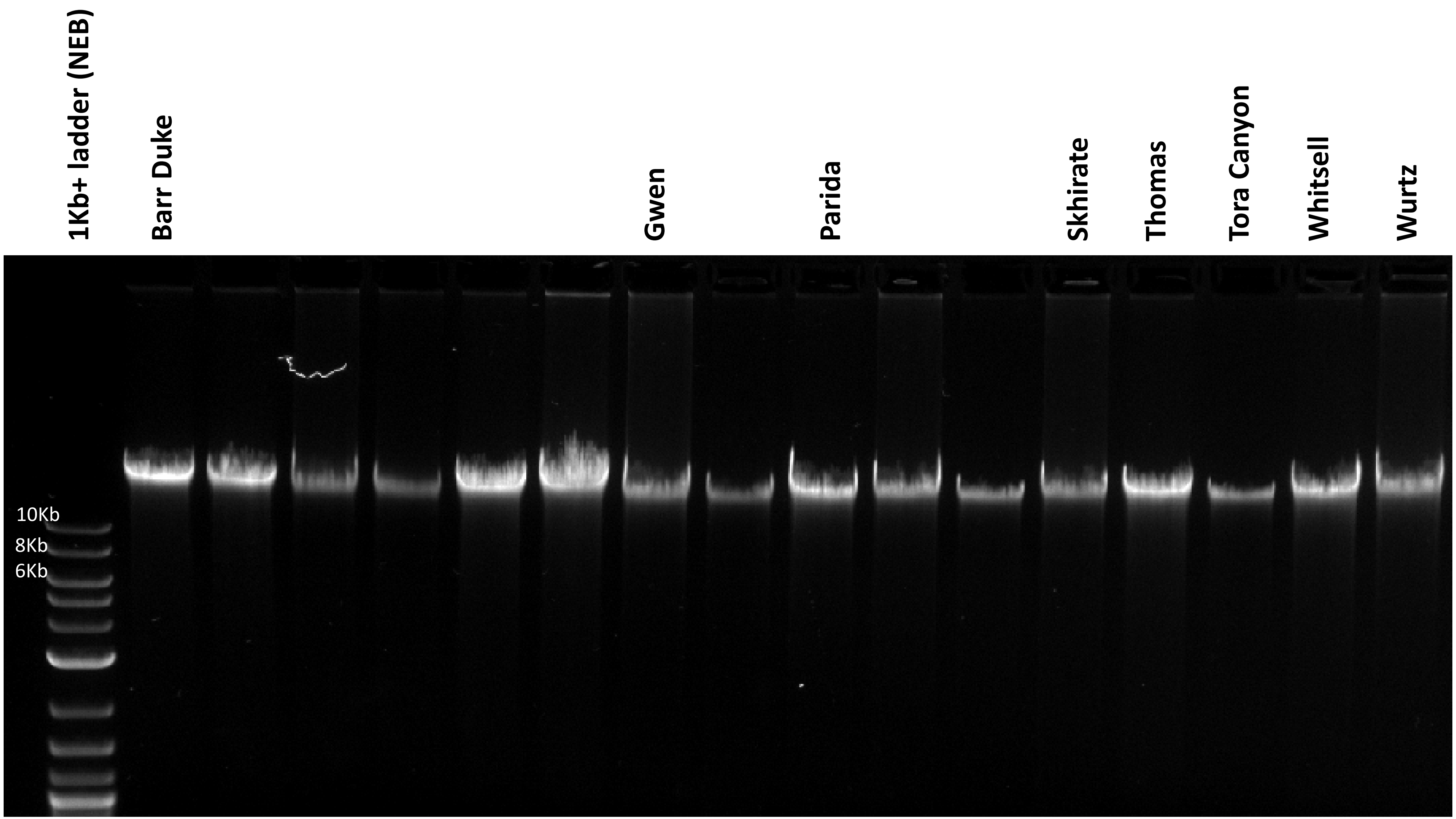

Figure S1A: Original Gel image corresponding to Figure 1-B indicating various cultivars in different lanes. The extracted DNA bands are intact and of high molecular weight as compared to 10kb band of 1kb+ ladder. 1µl extracted DNA was loaded on 1% agarose gel and ran at 90 volts for 45 minutes.

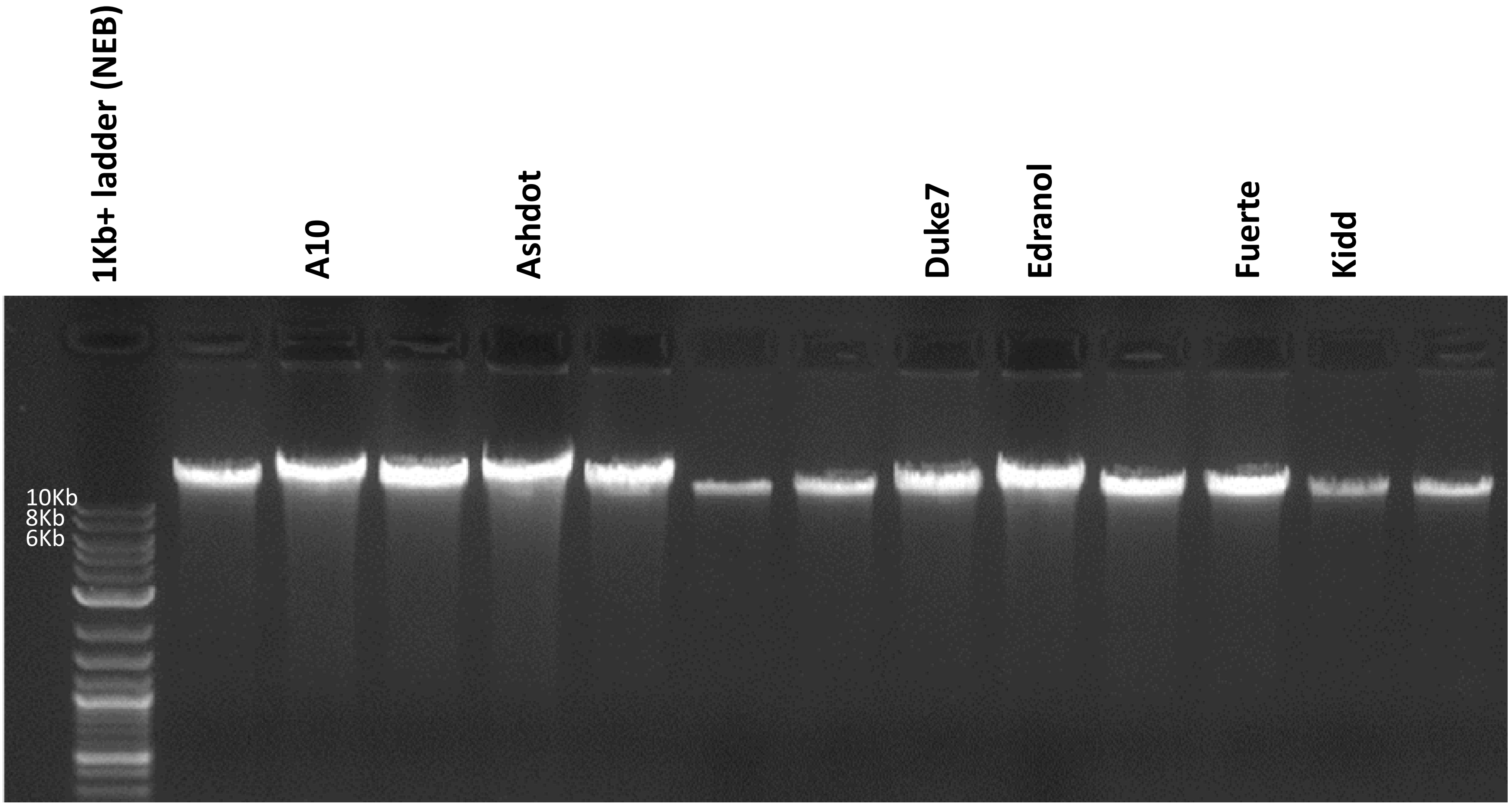

Figure S1B: Original Gel image corresponding to Figure 1-B indicating various cultivars in different lanes. The extracted DNA bands are intact and of high molecular weight as compared to 10kb band of 1kb+ ladder. 1µl extracted DNA was loaded on 1% agarose gel and ran at 90 volts for 45 minutes.

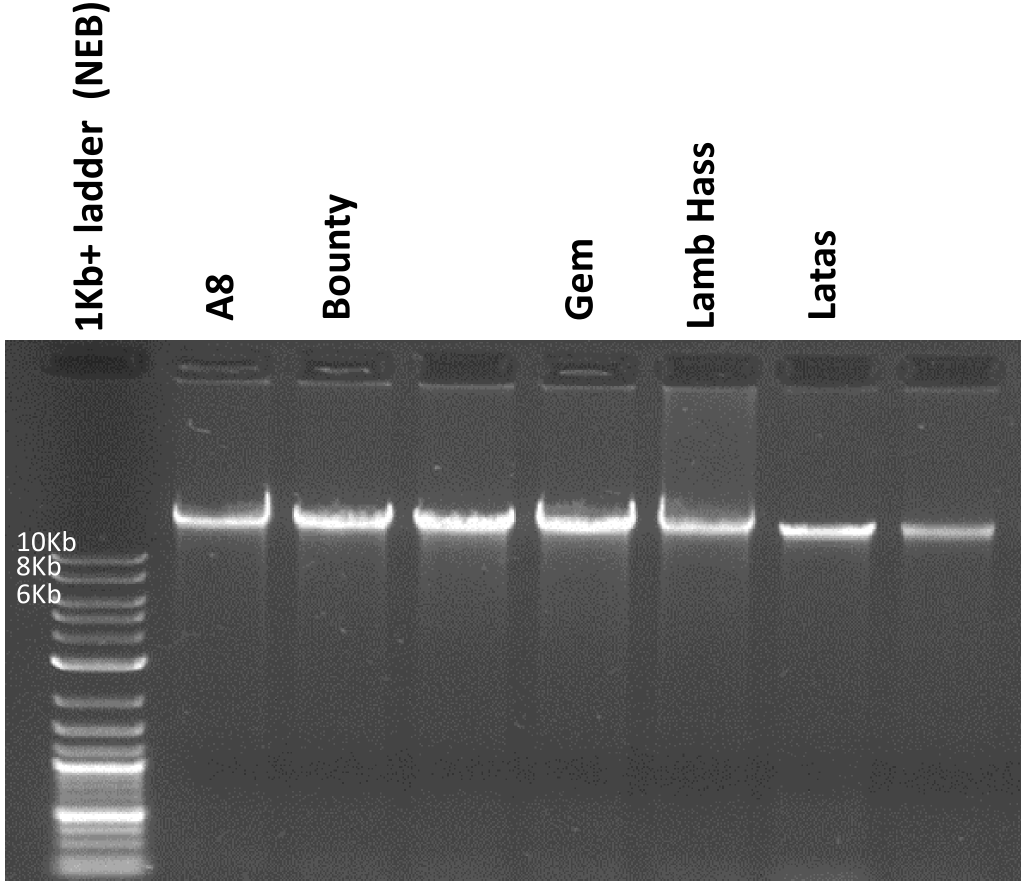

Figure S1C: Original Gel image corresponding to Figure 1-B indicating various cultivars in different lanes. The extracted DNA bands are intact and of high molecular weight as compared to 10kb band of 1kb+ ladder. 1µl extracted DNA was loaded on 1% agarose gel and ran at 90 volts for 45 minutes.

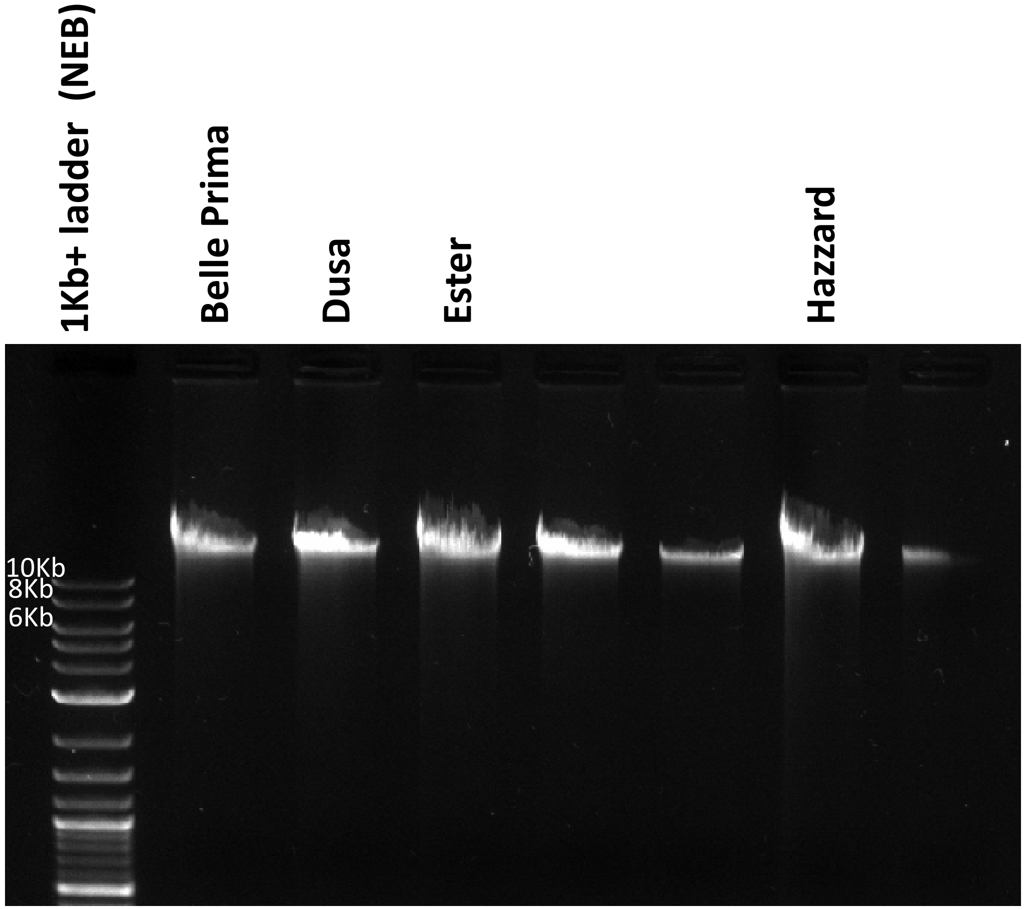

Figure S1D: Original Gel image corresponding to Figure 1-B indicating various cultivars in different lanes. The extracted DNA bands are intact and of high molecular weight as compared to 10kb band of 1kb+ ladder. 1µl extracted DNA was loaded on 1% agarose gel and ran at 90 volts for 45 minutes.

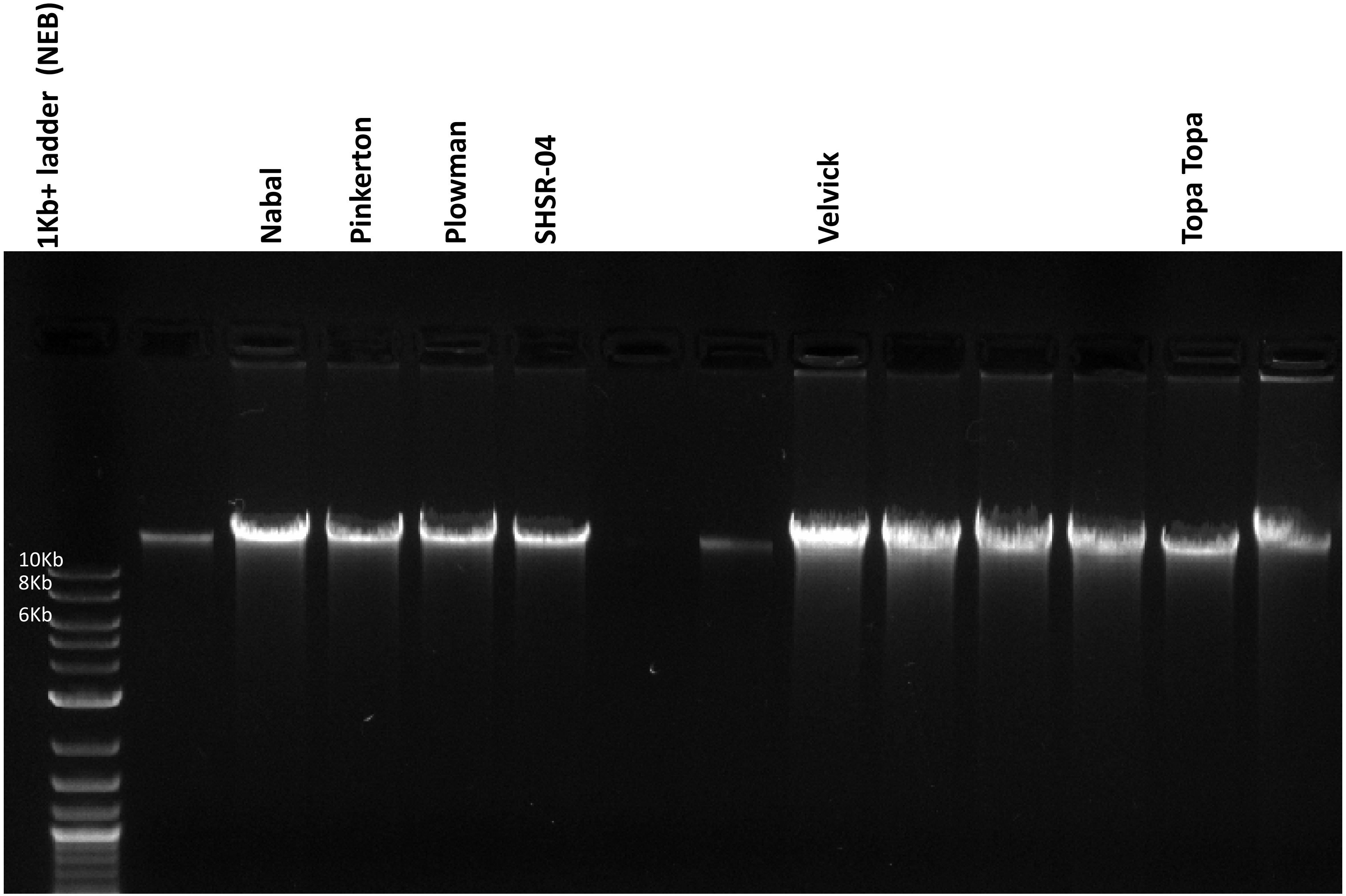

Figure S1E: Original Gel image corresponding to Figure 1-B indicating various cultivars in different lanes. The extracted DNA bands are intact and of high molecular weight as compared to 10kb band of 1kb+ ladder. 1µl extracted DNA was loaded on 1% agarose gel and ran at 90 volts for 45 minutes.

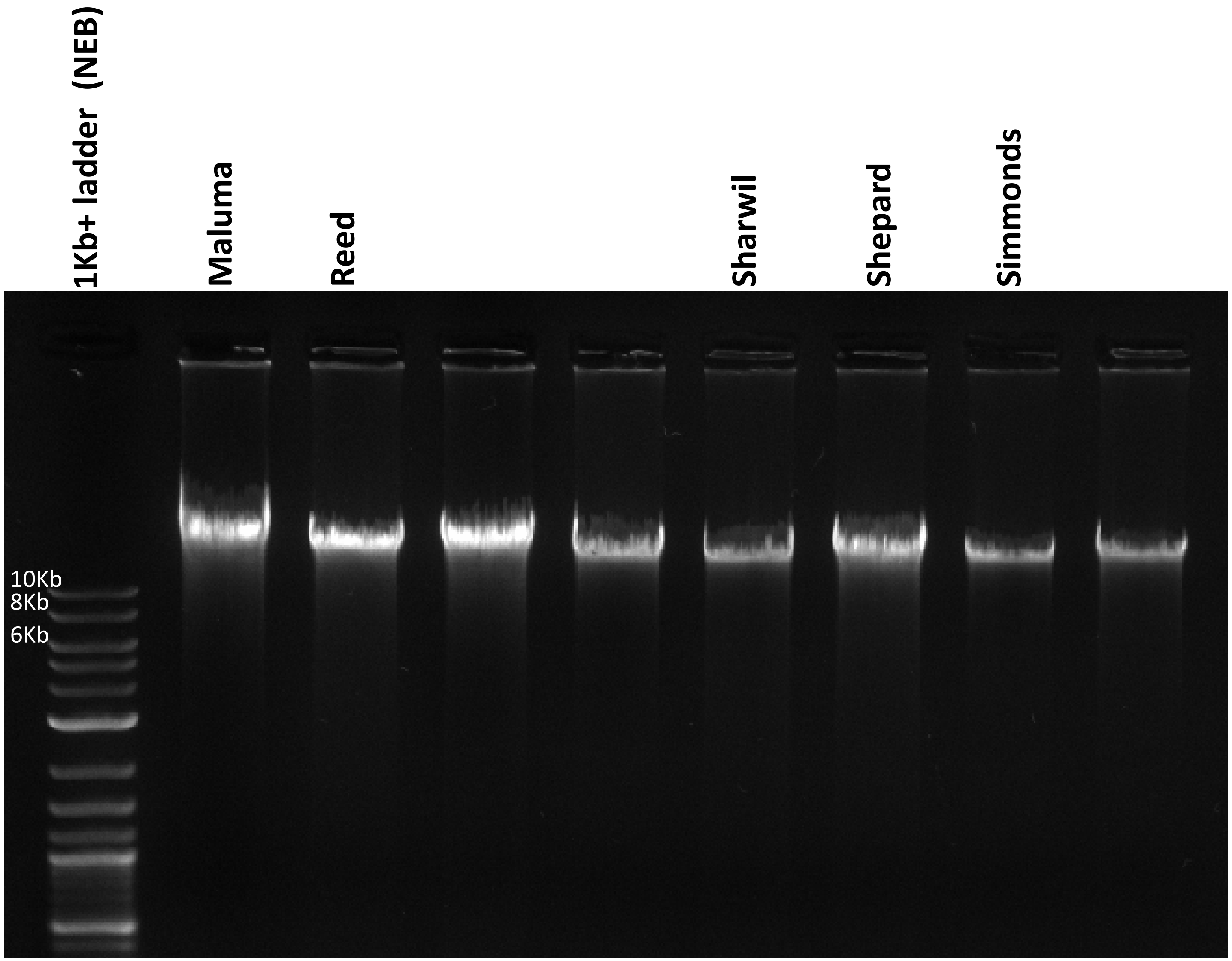

Figure S1F: Original Gel image corresponding to Figure 1-B indicating various cultivars in different lanes. The extracted DNA bands are intact and of high molecular weight as compared to 10kb band of 1kb+ ladder. 1µl extracted DNA was loaded on 1% agarose gel and ran at 90 volts for 45 minutes.

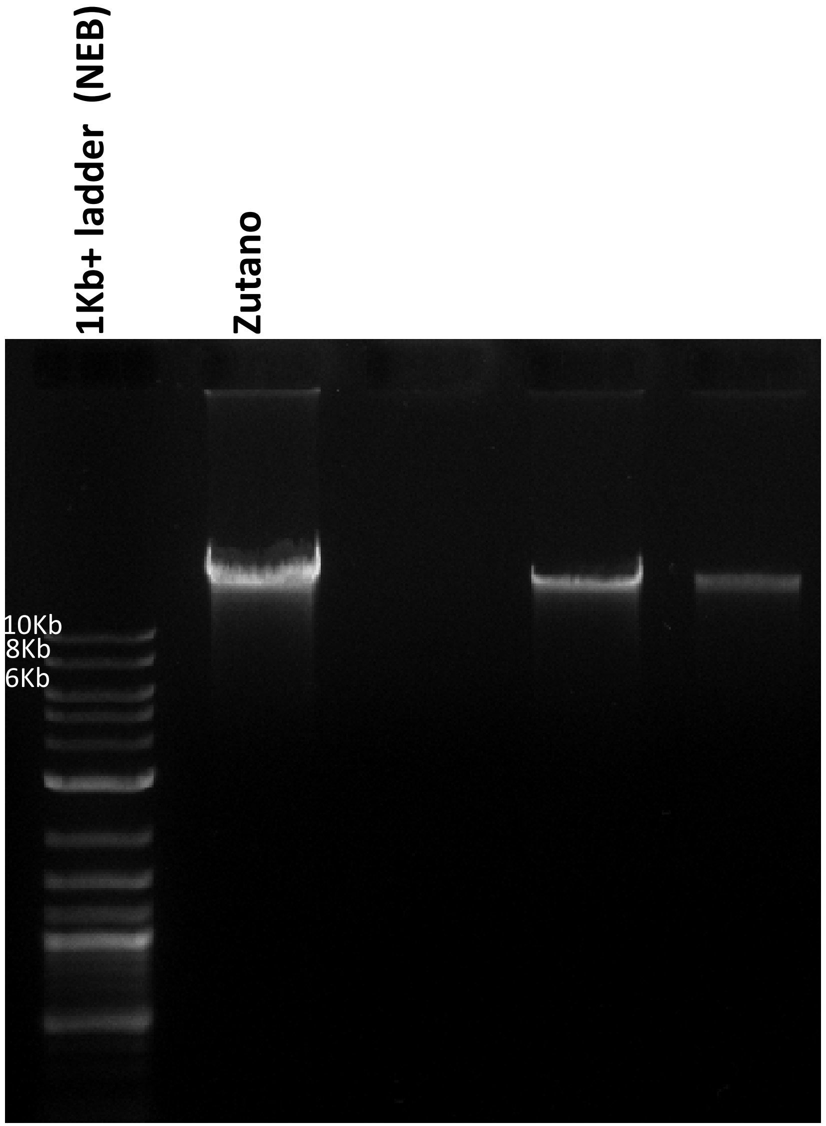

Figure S1G: Original Gel image corresponding to Figure 1-B indicating Zutano cultivar in second lane. The extracted DNA bands are intact and of high molecular weight as compared to 10kb band of 1kb+ ladder. 1µl extracted DNA was loaded on 1% agarose gel and ran at 90 volts for 45 minutes.

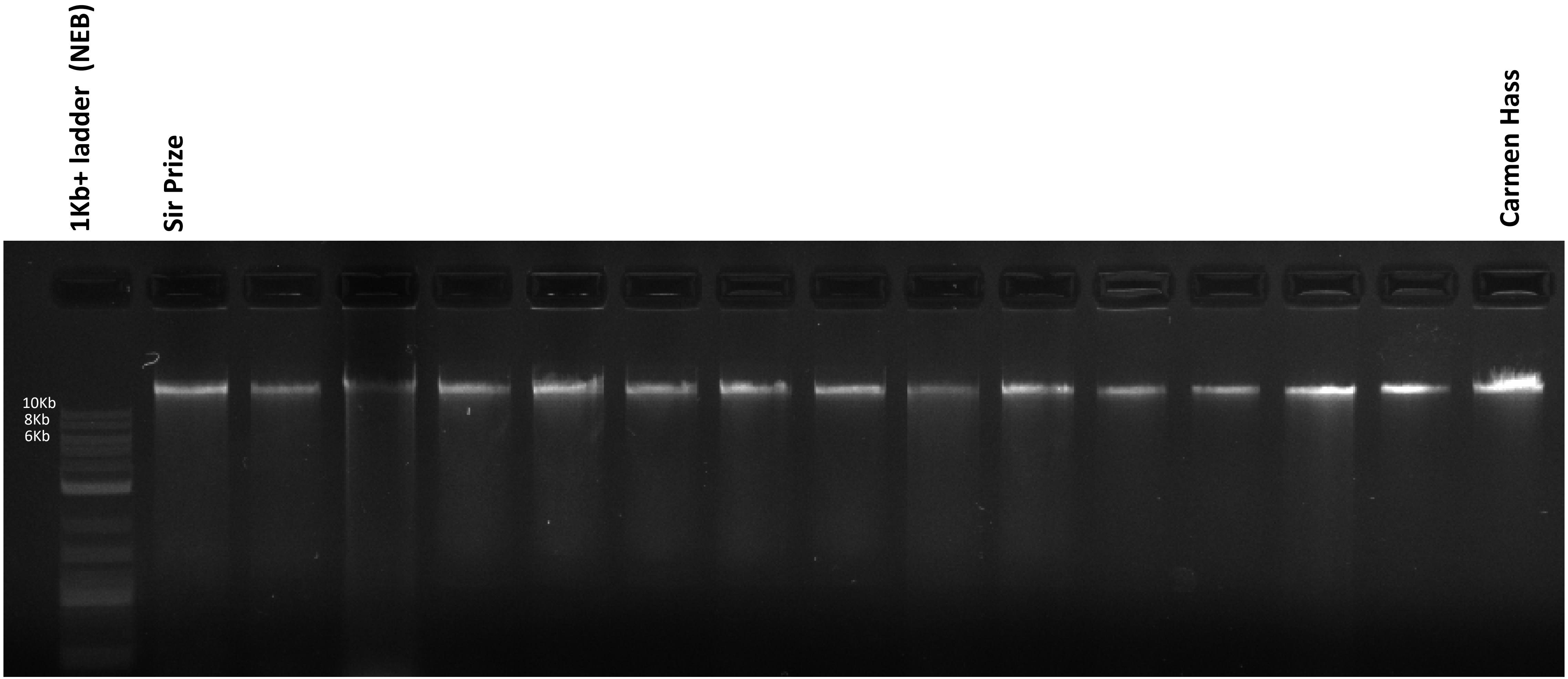

Figure S1H: Original Gel image corresponding to Figure 1-B indicating Sir Prize and Carmen Hass in 2nd and 16th lane respectively. The extracted DNA bands are intact and of high molecular weight as compared to 10kb band of 1kb+ ladder. 1µl extracted DNA was loaded on 1% agarose gel and ran at 90 volts for 45 minutes.

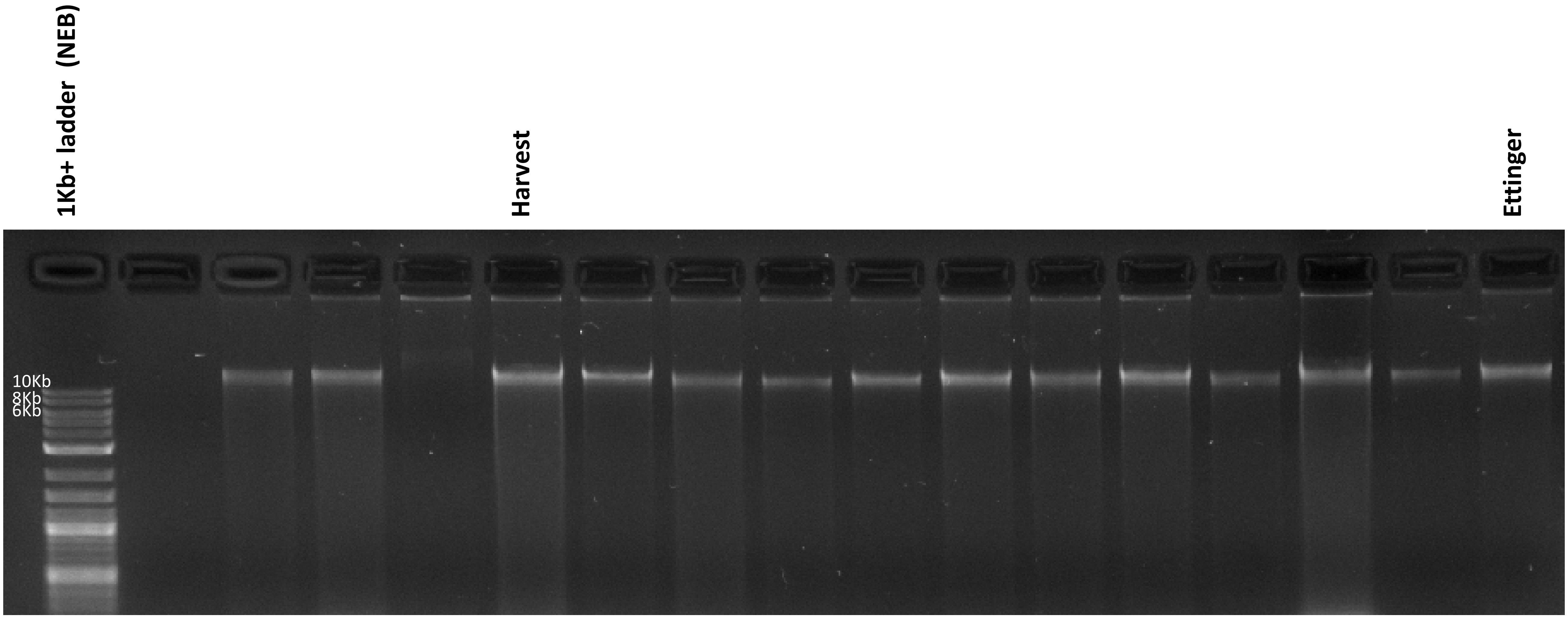

Figure S1I: Original Gel image corresponding to Figure 1-B indicating Harvest and Ettinger in 6th and 17th lane respectively. The extracted DNA bands are intact and of high molecular weight as compared to 10kb band of 1kb+ ladder. 1µl extracted DNA was loaded on 1% agarose gel and ran at 90 volts for 45 minutes.

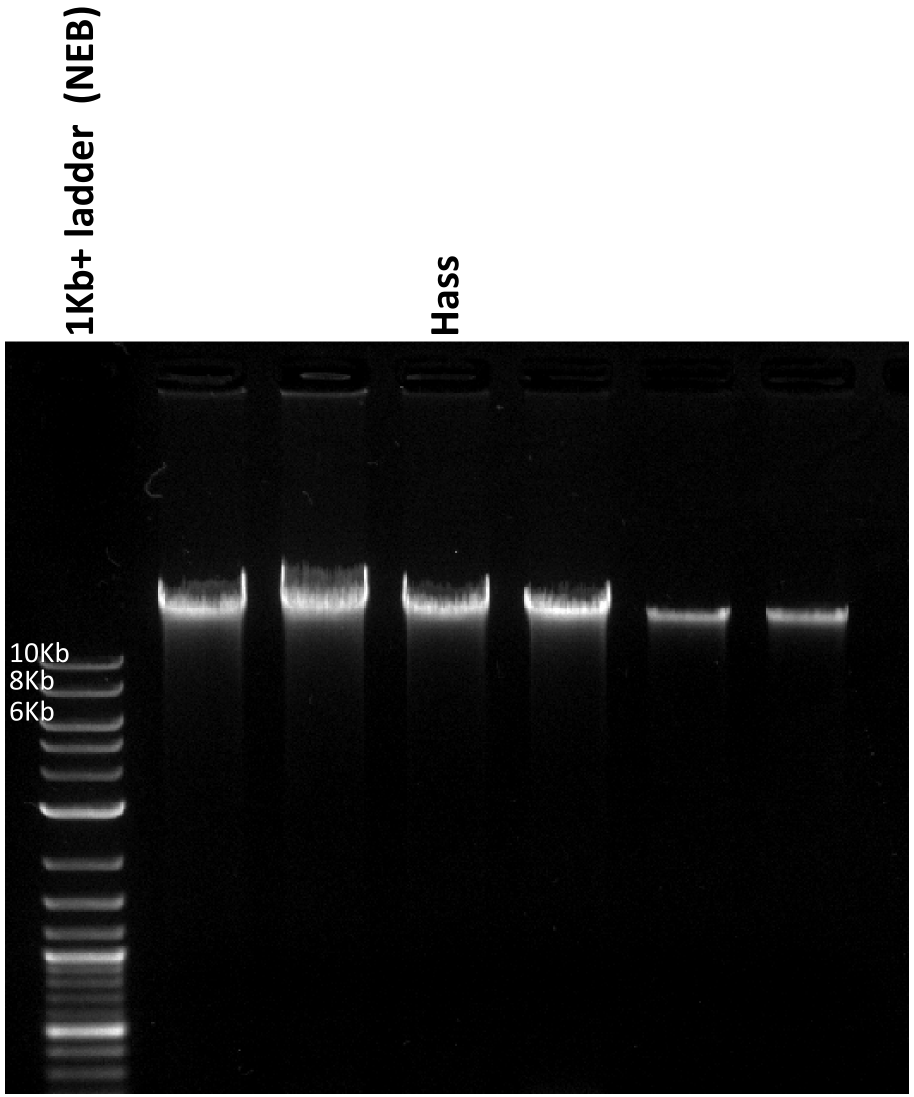

Figure S1J: Original Gel image corresponding to Figure 1-B indicating Hass cultivar in fourth lane. The extracted DNA bands are intact and of high molecular weight as compared to 10kb band of 1kb+ ladder. 1µl extracted DNA was loaded on 1% agarose gel and ran at 90 volts for 45 minutes.
